# Simulated Annealing-Based Image Reconstruction for Patients With COVID-19 as a Model for Ultralow-Dose Computed Tomography

**DOI:** 10.3389/fphys.2021.737233

**Published:** 2022-01-14

**Authors:** Shahzad Ahmad Qureshi, Aziz Ul Rehman, Adil Aslam Mir, Muhammad Rafique, Wazir Muhammad

**Affiliations:** ^1^Department of Computer and Information Sciences, Pakistan Institute of Engineering and Applied Sciences (PIEAS), Islamabad, Pakistan; ^2^Agri & Biophotonics Division, National Institute of Lasers and Optronics College, PIEAS, Islamabad, Pakistan; ^3^Department of Computer Engineering, Ankara Yıldırım Beyazıt University, Ankara, Turkey; ^4^Department of Computer Science and Information Technology, King Abdullah Campus Chatter Kalas, The University of Azad Jammu & Kashmir, Muzaffarabad, Pakistan; ^5^Department of Physics, King Abdullah Campus Chatter Kalas, The University of Azad Jammu & Kashmir, Muzaffarabad, Pakistan; ^6^Department of Physics, Charles E. Schmidt College of Science, Florida Atlantic University, Boca Raton, FL, United States

**Keywords:** inverse problem, simulated annealing, cost functions, COVID-19 patients, Radon transform, ultralow dose CT

## Abstract

The proposed algorithm of inverse problem of computed tomography (CT), using limited views, is based on stochastic techniques, namely simulated annealing (SA). The selection of an optimal cost function for SA-based image reconstruction is of prime importance. It can reduce annealing time, and also X-ray dose rate accompanying better image quality. In this paper, effectiveness of various cost functions, namely universal image quality index (UIQI), root-mean-squared error (RMSE), structural similarity index measure (SSIM), mean absolute error (MAE), relative squared error (RSE), relative absolute error (RAE), and root-mean-squared logarithmic error (RMSLE), has been critically analyzed and evaluated for ultralow-dose X-ray CT of patients with COVID-19. For sensitivity analysis of this ill-posed problem, the stochastically estimated images of lung phantom have been reconstructed. The cost function analysis in terms of computational and spatial complexity has been performed using image quality measures, namely peak signal-to-noise ratio (PSNR), Euclidean error (EuE), and weighted peak signal-to-noise ratio (WPSNR). It has been generalized for cost functions that RMSLE exhibits WPSNR of 64.33 ± 3.98 dB and 63.41 ± 2.88 dB for 8 × 8 and 16 × 16 lung phantoms, respectively, and it has been applied for actual CT-based image reconstruction of patients with COVID-19. We successfully reconstructed chest CT images of patients with COVID-19 using RMSLE with eighteen projections, a 10-fold reduction in radiation dose exposure. This approach will be suitable for accurate diagnosis of patients with COVID-19 having less immunity and sensitive to radiation dose.

## Introduction

The COVID-19, a pandemic declared since March 11, 2020, emerging from China, has seriously affected 213 countries and territories. According to WHO, mild symptoms have been observed in 80% of the patients with COVID-19 with mortality rate reported to be higher than 6% (Mahase, 2020). Immunity and radiation sensitivity during the COVID-19 diagnosis is the major challenge which is indirectly dependent on cost function selection for image reconstruction. The death toll till today is 3,955,972 with coronavirus reported cases as 1,82,686,233. COVID-19 affects the lungs, causing symptoms primarily such as pneumonia, resulting in diffused damage to both lungs of the patient necessitating the prompt action for its obliteration (Pan Y. et al., 2020). In this context, there are two alternatives for early diagnostic of COVID-19: first, real-time RT-PCR of viral nucleic acid in COVID-19 diagnosis having varying turnaround times with false-negative results, and second, the computed tomography (CT) imaging (Qureshi and Ul Rehman, 2020). The former approach has low sensitivity (59%) in comparison with the CT scan (88%) to diagnose the suspected subjects (Ai et al., 2020; Fang et al., 2020; Li and Xia, 2020). CT examination not only helps in diagnosis of COVID-19 disease but also in monitoring the development and evaluation of therapeutic efficacy. Ground glass opacities (GGO) bilateral distribution with or without consolidation in posterior and peripheral lungs is the cardinal hallmark of COVID-19 (Pan F. et al., 2020; Zhao et al., 2020). However, crazy paving pattern, airway changes, reversed halo sign, etc. (Dai W. C. et al., 2020; Smith et al., 2020; Ye et al., 2020) may shed light on the possible mechanism of lung injury in patients with COVID-19. For radiologists and other healthcare professionals, methods such as artificial intelligence-based volume segmentation may help by providing a faster way of objectively evaluating the radiological CT images.

Tomography refers to exposure of an object to the radiations in different orientations, and the transmitted data are used as an estimation of the object cross-section (Kak et al., 2002). The inversion problem for image reconstruction uses the numerical relationship between variations in a physical property in the area of interest. This is carried out by inverting the set of measurements, sinogram, with the approximation as the reconstructed image. It is conventionally estimated with filtered back-projection (FBP) that is accompanied by high dose rate with poor detectability as the absorption coefficient is low with other imperfections such as high noise and streak artifacts (Kak et al., 2002; Nelson et al., 2011). The risk prediction models for carcinogenesis (radiation-induced) have revealed that approximately 2% of the cancers in the US are thought to be linked with CT scans in a few decades (Brenner and Hall, 2007). General principles of dose reduction and optimization include adopting optimal number of projections, angle of projection (Sagara et al., 2010), tailoring a scan to a patient, minimizing scan length, minimizing tube voltage and current, iterative image reconstruction, and periodic review of CT studies (McCollough et al., 2009; Trattner et al., 2014). Landweber iterative algorithm is also used which calculates the quality of image reconstruction in terms of mean absolute error and correlation coefficient (Nagarajan and Kumar, 2020). Efforts have also been made to obtain better image quality using less number of projections with the help of various iterative reconstruction techniques (Southard et al., 2019) along with three-dimensional iterative image reconstruction (3D-IIR) to get acceptance in clinical setting (Ishikawa et al., 2020).

In children referred to emergency noncontrast head CT, iterative model reconstruction on average reduce 22% relative dose, compared with FBP, with significantly improved objective and subjective image quality (Elmokadem et al., 2019). Radiation exposure in patients can be reduced significantly (mean absorbed organ and effective doses were reduced by approximately 95%) using low-dose chest CT protocols and model-based iterative reconstruction (MBIR) algorithm while maintaining image quality for detecting round-shaped lung metastases (Kaasalainen et al., 2019). A 1-D wavelet transform-based multiscale image reconstruction technique was introduced by Bhatia et al. (1996) using FBP coefficients in expanded form with 1-D wavelet basis. They transformed complete set of projection views and it was much closer to the time domain. The significant reconstructed image quality degradation was reported when limited number of projections was transformed. Algebraic reconstruction technique is an iterative way to estimate the cross-section at the cost of computational time (Herman, 2009). Expectation maximization (EM) technique has been used as a stochastic approach and its success is attributed to the reduction in dose rate as compared to FBP while resulting in comparable image quality with lesser number of projections (Smith-Bindman et al., 2010; Schindera et al., 2013). In this way, the dose rate to patient can be reduced many folds as its relationship with number of projections is assumed to be linear (Chen et al., 2008; Liu et al., 2012). Gjesteby et al. (2017) integrated the convolution neural network (CNN) into CT image reconstruction process. Instead of using a traditional stopping rule (threshold or maximum number of iterations) during iterative reconstruction, this study monitors the quality of CT image and decided to stop the process according to an intelligent numerical observer. For low-dose computed tomography (LDCT) scan, the iterative reconstruction results in degradation of image quality. To overcome these limitations, deep learning image reconstruction maintains the image quality and also reduction in dose. Kim et al. (2021) assessed the quality of image and noise of LDCT scan images which are reconstructed with deep learning image reconstruction. In low-dose X-ray CT, severe artifacts typically occur due to photon starvation, beam hardening, and other causes, all of which decrease the reliability of the diagnosis. Kang et al. (2017) proposed an algorithm which uses a deep convolutional neural network (CNN) which is applied to the wavelet transform coefficients of low-dose CT images. Similarly, Lee et al. (2018) proposed a deep-neural-network-enabled sinogram synthesis method for sparse-view CT. The proposed network produced promising results and is believed to play an important role as an option to the low-dose CT imaging.

The Metropolis criterion (Kirkpatrick et al., 1983) is used as the basis in the simulated annealing (SA) where the global optimization stages are traversed in the search space to avoid local optima. In case of SA, the decision to accept the change is independent of the cost incurred for new change so it can be easily converged (Granville et al., 1994). This algorithm has been used in numerous applications such as phase measurement profilometry (Dai M. et al., 2020), bone material identification (Wilkie et al., 2020), analog circuit design, piezoelectric device optimization, core loading pattern optimization in nuclear reactors, communication code design, image restoration, traveling sales man problem, and identification of military targets without experts (Geman and Geman, 1984; Gamal et al., 1987; Johnson et al., 1989; Pai and Sreeram, 2002; Vetterling et al., 2002; Han and Chatterjee, 2004; Zameer et al., 2014). Haneishi et al. (1990) applied SA to reconstruction of CT images and found that the modified cost function is unavoidable to suppress artifacts originating due to the ambiguity of null components. Greening (1994, 1995) and Smith et al. (2008) found that the selection of appropriate cost function for SA not only affects the convergence rate but may also lead to inaccuracies with degraded outcome. The determination of an optimal cost function for SA-based image reconstruction still needs to be considered to reduce the patient dose rate as it uses lesser number of projections. Candès et al. (2006) algorithm used incomplete projections for image reconstruction by compressive sensing technique and proved substantial reconstructed image quality. In comparison, their method cannot be compared with stochastic methods due to the inherent differences lying in their mathematical foundations. Although, the modern days GPU-based systems can involve massive parallelism to counter the time lapse involved in the stochastic problem-solving strategies.

Particle swarm optimization (PSO), genetic algorithm (GA), and SA belong to the class of stochastic processes. Each of these methods has its own merits and demerits. Researchers have worked in each of the specific domains for their problems keeping in view the problem structure and specific parameters and graded them in multiple ways (Rajendra and Pratihar, 2011; Jia and Lichti, 2017), and the fact is that the inherent working of each of the methods is different. The results obtained by GA and those by PSO have been compared, and the performance of latter has been found relatively better, as the PSO carries out global search and local searches simultaneously whereas the GA concentrates mainly on the global search (Rajendra and Pratihar, 2011). In addition to this, GA is slower in the final convergence stage due to loss of diversity whereas PSO is relatively faster in this respect. PSO easily falls into local optima problem in case of high-dimensional space whereas GA solves complex optimization problems with artificial intelligence approach. The program simplicity is more in case of PSO whereas GA, with unguided mutation and computational expense, is challenging in finding an objective function with appropriate representation and suitable operators working in line with the Darwin’s theory of evolution and mutation philosophy. In this context, SA uses different cost functions while dealing with arbitrary and complex systems to find an optimal solution that is statistically guaranteed (Lecchini-Visintini et al., 2007). It is a search algorithm based on a solo-sequence, simulating the physical process of cooling metals while furnace cooling or annealing to acquire an optimized solution. Some researchers have introduced hybrid diversification operators using SA in GA to solve the problem of diversity loss for image quality optimization problem (Qureshi et al., 2011). The proposed model focusses on image reconstruction using numerous cost functions at a low-dose rate, using incomplete or missing CT projections, to reconstruct high-quality images.

As the early diagnosis of COVID-19 is essential, the reduced dose rate is highly desirable when CT scan is conducted, especially in case of multiple scans due to destructive nature of ionizing radiations being used as viewing source. Even biomolecules signatures (Rehman and Qureshi, 2020) produced can be used for virus replication process in the alveoli but still CT imaging is the authentic technique to diagnose the COVID-19 disease (Ul Rehman and Qureshi, 2020). Therefore, CT image reconstruction using an ultralow dose rate-based SA with a suitable cost function is desirable to solve this problem. In this work, the cost functions, namely universal image quality index (UIQI), root-mean-squared error (RMSE), structural similarity index measure (SSIM), mean absolute error (MAE), relative squared error (RSE), relative absolute error (RAE), and root-mean-squared logarithmic error (RMSLE) have been evaluated and critically analyzed for ultralow dose rate image reconstruction. Their annealing and execution times in case of 8 × 8 and 16 × 16 lung phantoms have been described and compared. RMSLE after optimizing with lung phantom has been successfully implemented on patients with COVID-19 and their image reconstruction has been performed with 18 projections achieving an ultralow dose for patients with COVID-19 and compared with actual patient’s CT images.

## Materials and Methods

**FIGURE 1:**
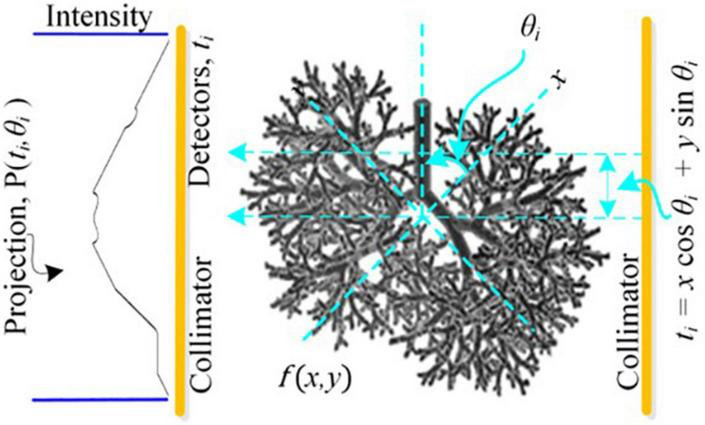
Model depicting forward projection at an orthogonal distance *t* through the center of a hypothetical cross-section *f*(*x*,*y*) of lung rotated by θ in Cartesian coordinates (*x*,*y*).

**FIGURE 2:**
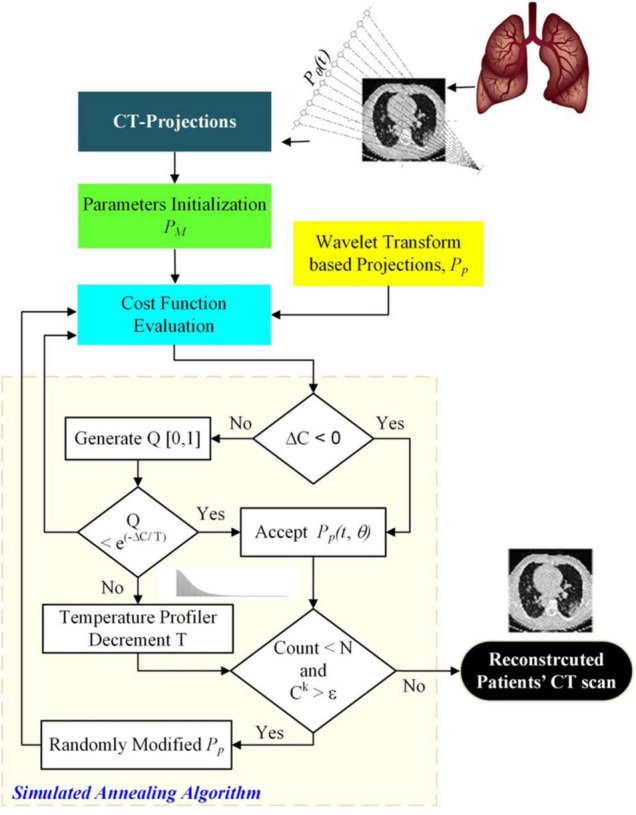
Ultralow Dose CT-based image reconstruction of COVID-19 patient’s lungs using simulated annealing.

The image reconstruction has four basic parts: a cost function that finds the misfit between the measured and postulated projections; the Metropolis criteria that randomly accepts the solution in case the cost is high; a set of generic parameters, namely initial and final temperatures; and problem-specific parameters (PSPs). For the CT reconstruction problem, the important PSPs are view angles, number of projections, and image size. In parallel ray transmission tomography, the projection for a view angle θ can be obtained by measuring the transmitted intensities through an object f(x,y) as shown in the model in Figure 1. The resorting algorithm transforms the fan beam data into the equivalent parallel beam data which can be used for image reconstruction (Kak et al., 2002). The proposed methodology for the ultralow-dose CT image reconstruction is shown in Figure 2.

The RT, an integral transform, is a discrete sequential line integral for a specific angular view using digital image like spatial grid (or matrix) distributed within the object across a slice. For a single projection P_θ_(t), along (θ,*t*)-line, a Radon transform is given by (Buzug, 2011):


(1) $$\text{P\_θ}\,\left(t\right)=\int_{-\infty }^{\infty }\int_{-\infty }^{\infty }\text{f}\,\left(x,y\right)\,\delta \,\left(x\,\cos\theta +\text{y}\,\sin\theta -t\right)\,\mathrm{dx}\,\mathrm{dy},$$

-∞<t<∞, 0≤θ<π.

This is an inverse problem where the unknown function f(x, y) is computed using X-ray-based sensors-acquired set of projections P_θ_ (t). Bhatia et al. (1996) used multiscale data filter matrix R_W_ = WR_fbp_W^T^ using Daubechies or Haar wavelet family where R_fbp_ is the ramp filter of the conventional FBP technique. The 1-D wavelet transform of actual projections results in θ^th^ multiscale projection η_θ_ = WP_θ_ and the filtered projections are given by ξ_θ_ = R_W_η_θ_. We used universal thresholding scheme (Donoho and Johnstone, 1994; Yao and Rajpoot, 2005) where all wavelet coefficients higher than a threshold are used and the remaining are removed (Scheunders, 2004) and these coefficients are back-projected afterward along the θ^th^ multiscale basis function. The overall estimate of back-projection process over B number of detector bins is given by: f^WT⁢(x,y)=∑θ=0MTθT⁢ξθ, where T_θ_ represents M × B^2^ matrix for viewing angle θ and M represents viewing angles count. The approximated IRT, ${\hat{\text{f}}}_{\text{WT}}$, is used as a template to initialize the population. The sinogram or the measured projections P_M_(t,θ) for multiple views, by varyingθ, is collected through the data acquisition and control system of CT machine. The linear Radon transform is based on the summation of absorption coefficients that are distributed in a highly nonlinear manner. Its discrete representation as a forward problem is given by:


(2) $${\text{P}}_{\text{M}}\,\left(t,\theta \right)=\sum_{x=0}^{{\text{r}}_{\text{m}}-1}\sum_{y=0}^{{\text{c}}_{\text{m}}-1}\sum_{\theta =0}^{\theta_{\text{n}}}\text{f}\,\left(x,y\right)\,\mu \,\left(x,y\right)$$

$$\mu \,\left(x,y\right)=\left\{ \begin{array}{cc} 1 & \mathrm{if}\,t=x\,\cos\theta +y\,\sin\theta \\ 0 & \text{otherwise} \end{array}\right.$$

Here, r_m_×c_m_ forms the cross-section to be estimated and μ(x,y) represents the distribution of absorption coefficients. The postulated projectionsP_P_ (t,) are generated by randomly changing the pixel intensity for the IRT approximation. The energy or cost function corresponding to the kth iteration, Ck, is given in Section “Pseudocode for the Proposed Algorithm.” The objective is to minimize the error between the measured and postulated projections using numerous cost functions under different constraints, consisting of CT-specific (PSP) and generic parameters of SA. The simulations are carried out repeatedly to reach a lower temperature state along with optimization of image reconstruction quality. The postulated projections, corresponding to the estimated IRT, ${\hat{\text{f}}}_{\text{k}}$ with an error $△\,\hat{\text{f}}$, are obtained in an iterative manner *via* forward projections as ${\hat{\text{f}}}_{k+1}$. The estimated image, without using any backward projections, is given by ${\hat{\text{f}}}_{k+1}=△\,\hat{\text{f}}+{\hat{\text{f}}}_{\text{k}}$. The regularization to get a finite and meaningful solution is solved by generating random numbers for sampling position in the image domain and its quantized intensity level in the range [0, 255]. Let △C indicates the cost variation as given by △C=C_k+1_−C_k_, where C_k+1_ andC_k_represent the cost of ${\hat{\text{f}}}_{k+1}$and ${\hat{\text{f}}}_{\text{k}}$, respectively. The change ${\hat{\text{f}}}_{k+1}$is admitted for the iteration if the condition △C < 0 is TRUE. The acceptance probability, for the kth iteration, ${\hat{\text{f}}}_{k+1}$, in case △C < 0 is FALSE, is given by:


(3) $${\text{h}}_{\text{k}}=\exp\,\left(\frac{-△\,\text{C}}{{\text{T}}_{\text{k}}}\right)$$

Here, T_*k*_ is the annealing temperature for the kth iteration. Equation 3 shows that the decrease in temperature is accompanied by a corresponding improvement in acceptance occurrences for the worst-case scenarios. The possibility of acceptance of a variation at even higher cost prevents the algorithm from local minima trapping. Various cost functions experimented for ultralow-dose CT image reconstruction using SA are presented in Table 1. Regarding the mathematical notation employed in this paper, Table 2 describes the main symbols used to designate the data items and operations, among others. The pseudocode for the proposed algorithm follows next.

**TABLE 1:** Cost functions used for image reconstruction and their mathematical relationship for θ^th^ view.

Cost function	References	Relationship
UIQI	Wang and Bovik, 2002	$U\,I\,Q\,I^{\theta }=\frac{\sigma_{P_{M}}{}_{P_{P}}}{\sigma_{P_{M}}\,\sigma_{P_{P}}}.\frac{2\,\bar{P_{M}\,P_{P}}}{{\bar{P_{M}}}^{2}+{\bar{P_{P}}}^{2}}.\frac{2\sigma_{P_{M}}{}_{P_{P}}}{\sigma_{P_{M}}^{2}+\sigma_{P_{P}}^{2}}$
RSE	Arnold and Stahlecker, 2002	$R\,S\,E^{\theta }=\frac{\sum_{t=0}^{t_{b}-1}{\left(P_{M}\,\left(t\right)-P_{P}\,\left(t\right)\right)}^{2}}{\sum_{t=0}^{t_{b}-1}{\left(\bar{P_{M}}-P_{M}\,\left(t\right)\right)}^{2}}$
SSIM	Wang et al., 2004	*S^θ^(P*_*M*_,*P*_*P*_) = *f*(*l*(*P*_*M*_,*P*_*P*_),*c*(*P*_*M*_,*P*_*P*_),*s*(*P*_*M*_,*P*_*P*_))
MAE	Hyndman, 2006	$M\,A\,E^{\theta }=\frac{1}{t_{b}}\,\sum_{t=0}^{t_{b}-1}\left|P_{M}\,\left(t\right)-P_{P}\,\left(t\right)\right|$
RAE	Zomaya and Kazman, 2010	$R\,A\,E^{\theta }=\frac{\sum_{t=0}^{t_{b}-1}\left|P_{M}\,\left(t\right)-P_{P}\,\left(t\right)\right|}{\sum_{t=0}^{t_{b}-1}\left|\bar{P_{M}}-P_{M}\,\left(t\right)\right|}$
RMSE	Liu et al., 2017	$R\,M\,S\,E^{\theta }=\sqrt{\frac{1}{t_{b}}\,\sum_{t=0}^{t_{b}-1}{\left(P_{M}\,\left(t\right)-P_{P}\,\left(t\right)\right)}^{2}}$
RMSLE	Leke and Marwala, 2019	$R\,M\,S\,L\,E^{\theta }=\sqrt{\frac{1}{t_{b}}\,\sum_{t=0}^{t_{b}-1}{\left(l\,o\,g\,\left(P_{M}\,\left(t\right)-P_{P}\,\left(t\right)\right)\right)}^{2}}$

**TABLE 2:** Mathematical notation summary for proposed low-dose CT-based image reconstruction system.

Symbols	Meanings
*t*_*b*_	Total number of detector bins
${\hat{f}}_{W\,T}$	Wavelet transform-based approximated IRT
*C^k^*	Cost (or energy) function for kth iteration
△*C*	Cost variation in simulated annealing
${\hat{f}}_{k}$	Estimated inverse Radon transform
$△\,\hat{f}$	Error in consecutive projections
*h_k*	Acceptance probability for kth iteration
*l*_*s*_	Length of the side of template
*N*	Total number of iterations for simulated annealing
*P*	Uniformly distributed projections over the interval [0, π]
*P*_θ_	Single projection along θ-view
*P*_*p*_	Postulated projections
*P*_*M*_	Measured projections
*R*_*fbp*_	FBP-based ramp filter matrix
*R_W*	FBP-based multiscale filter
*r_*m*_ × c_*m*_*	Cross-section to be estimated
*T*_0_	Initial annealing temperature
*T*_*f*_	Final annealing temperature
*T_k*	Annealing temperature for kth iteration
*W*	Matrix of the discrete 1-D wavelet transform operation
	1-D wavelet transform of projection θ
*μ(x, y)*	Absorption coefficients distribution
_*P_MP_P*_	Covariance between measured and postulated projections
_*P_M*_	Standard deviation of measured projections
_*P_P*_	Standard deviation of postulated projections
$\bar{P_{M}}$	Mean of measured projections
$\bar{P_{P}}$	Mean of postulated projections
*l*(*P*_*M*_,*P*_*P*_)	Luminance comparison function
*c*(*P*_*M*_,*P*_*P*_)	Contrast comparison function
*s*(*P*_*M*_,*P*_*P*_)	Structure comparison function
*T*	Bin number of detector
θ	Viewing angle
ξ	Filtered projection as 1-D wavelet transform

### Pseudocode for the Proposed Algorithm

**FIGURE 3:**
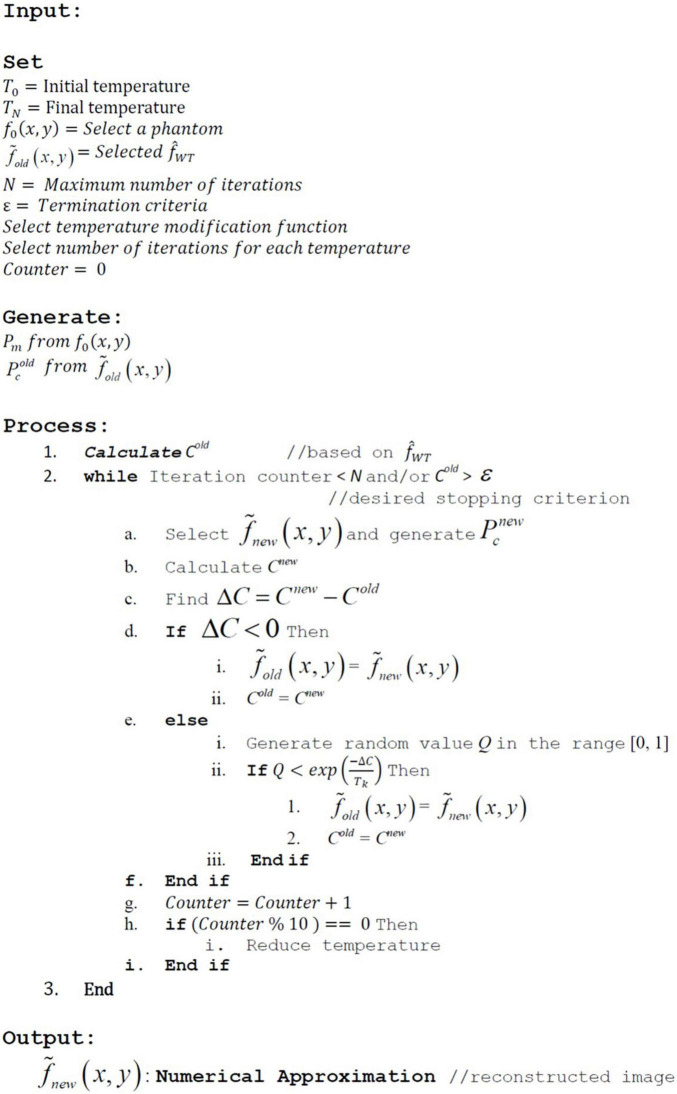
Pseudocode for the image reconstruction algorithm.

The selection of template, number of projections, size of IRT, and the random selection of gray levels according to some predefined criteria are the important variants that are addressed in the pseudocode illustrated in Figure 3. In addition to this, the SA parameters include the initial temperature, the final temperature, the annealing profile, and the number of iterations by which the temperature is kept constant.

### Cost Functions and Their Formulas

The cost functions, namely UIQI, RMSE, SSIM, MAE, RSE, RAE, and RMSLE, have been analyzed for image reconstruction.

### Wavelet Transform-Based Initializing Template

The lung phantom data have been used from the Institute of Medical Physics Friedrich-Alexander-University Erlangen-Nürnberg, Germany. It is a fractal model of the bronchial tree during which every branch of the lung phantom is terminated by a semisphere (hollow) connected *via* a cylinder (hollow) followed throughout. Out of one end of the branch grows two sprouts, a small one and a large one, during which lie the two sprouts are known as branch plane. The branch plane is revolved from generation to generation by a given angle to extent the structure from 2 to 3 dimensions and therefore to homogeneously fill a given volume (Fishburn et al., 1997; Vazquez-Corral et al., 2020).

The initial guess or templates for the starting image in SA is important as it considerably lowers the annealing time required for convergence according to a predefined criterion. In the primitive technique, namely back-projection, the matrix is projected backward or inverted along the corresponding degree of rotation between [0, π]. The initialization template is based on wavelet transforms to address the blurring effect in the spatial domain during back-projection resulting by inversion of Radon transforms. The multiscale data filter with Daubechies wavelet family is used on the conventional back-projection filter. The actual projections are subjected to a 1-D wavelet transform, and the multiscale projections for the specific geometrical view are multiplied with the wavelet transform of the R_*fbp*_ (ramp filter). The wavelet coefficients higher than a critical value are used with the leftover removed. These coefficients are back-projected to generate the IRT estimate.

**FIGURE 4:**
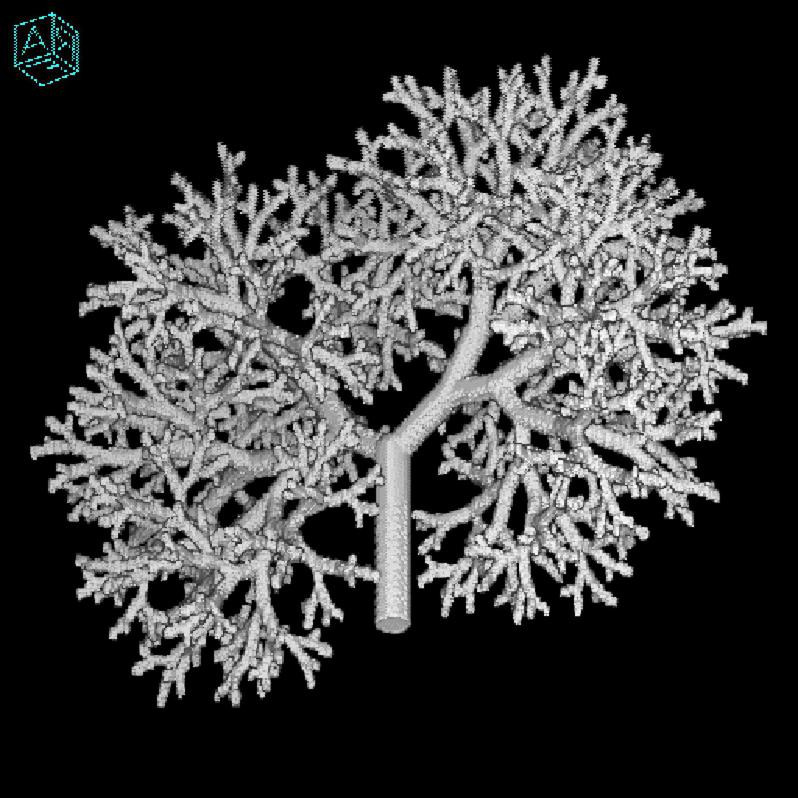
The 512 × 512 pixels lung phantom.

The wavelet transform-based image reconstruction method has been used to simulate 8 × 8- and 16 × 16-sized templates (${\hat{\text{f}}}_{\text{WT}}$) for postulated projections P_*p*_. The phantom used for simulation of measured projections PM is shown in Figure 4. The formal restraints imposed for uniform basis are same data size (512 × 512) for lung phantom, 512 collimator openings uniformly distributed, linear interpolation during back-projection, and zero noise level (Qureshi et al., 2007).

The optimized parameter set has been used for simulations, namely number of projections p=18 (uniformly distributed) which reduces the dose by several tens or even over hundred folds as compared to that in current CT practice. Initial temperature is kept T_0_ = 0.1 along with a uniform temperature slab thickness of 10^3^. Annealing times N = 2×10^5^ for 8 × 8- and N=8×10^5^ for 16 × 16-sized images have been used in all the simulations. Final temperature has been set as T_N_=1.5×10^−3^. The temperature profile used for kth iteration for lung phantom is directed by:


(4) $${\text{T}}_{\text{k}}=\frac{{\text{T}}_{0}-{\text{T}}_{\text{N}}}{\cosh\left(\frac{10\,k}{\text{N}}\right)\,{\text{T}}_{\text{N}}}.$$

The selection rule is set to iteration-count limit or the allowed variation in iterative cost function as 0.1. All the simulations have been based on 20 repetitions for each size of reconstructed image.

### COVID-19-Confirmed Patients’ Data

Once it was established that RMSLE produced agreeable results for lung phantom, it was implemented on COVID-19-confirmed patients’ data publicly available that have been taken from Italian Society of Medical and Interventional Radiology (SIRM, 2021).

### Performance Measures and Hardware

The reconstruction models and corresponding simulations have been carried out using standard software tools. The computation has been carried out using Dell Inspiron 5520 (processor: Intel(R) Core(TM) i7-3612QM CPU @ 2.1 GHz, generation 3) with 8 GB RAM, and image quality measures, namely PSNR, EuE, and WPSNR, have been used to assess the reconstruction class (Guo et al., 2020; Sohrabi et al., 2020).

## Experimental Results and Discussion

We have used lung phantom (Figure 4) to simulate the measured projections (P_*m*_) that are evenly distributed in the range [0, π], with zero noise level and standard deviation, having 256 intensity levels in the range [0, 1]. The postulated projections (P_*p*_) are simulated using the wavelet transform-based template, ${\hat{\text{f}}}_{\text{WT}}$, under the same prevailing conditions. The error between the measured and postulated projections is computed using the cost functions, namely UIQI, RMSE, SSIM, MAE, RSE, RAE, and RMSLE and this forms the basis of their comparison for their efficacy in image reconstruction through SA algorithm. The wavelet transform-based template is then modified iteratively according to the Metropolis criterion, and the changes in reconstructed image intensity are accepted or rejected accordingly (Kirkpatrick et al., 1983). The generic and CT-related parameters remain the same for whole cost function analysis. This SA technique has been used by many researchers to solve problems lying in similar domains. Ortiz-Alemán and Martin (2005) have put forward the solution of inversion problem using SA with poor templates for electrical capacitance tomography data with slow convergence rates, resulting in highly accurate images as compared to the traditional linear methods. Carletti et al. (2006) reported outstanding reconstruction with the SA-based algorithm for the electron beam spectrum. The SA was also used to reconstruct positron emission tomography (PET) images by Yaqub et al. (2006), and the results were found to be superior to the interior-reflective Newton method with no biasing. Webb (1989) had already introduced SA-based single photon emission computed tomography (SPECT) using the data acquired through gamma camera. A comparison of various forms of SA may be seen in detail by Ingber (1993).

**FIGURE 5:**
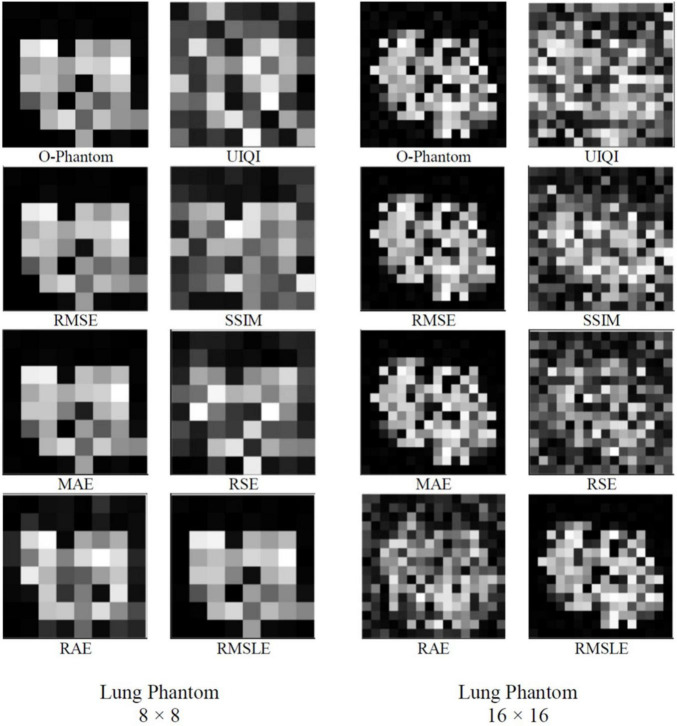
Comparison between 8 × 8 and 16 × 16-sized image reconstruction using simulated annealing for the lung phantom, by using original phantom image, and cost functions (UIQI, RMSE, SSIM, MAE, RSE, RAE, and RMSLE) (*p* = 18, *T*_*0*_ = 0.1, *T*_*N*_ = ×10^−6^, *N* = 8×10^5^, temperature slab thickness set to 1000, and temperature profile as given by Eq. 4).

A tabular comparison of numerous cost functions for SA on the basis of PSNR, EuE, and WPSNR resulting in 8 × 8 and 16 × 16 lung phantom images has been illustrated in Table 3. The sensitivity analysis of cost functions reveals that RMSLE has been found relatively more effective. The reconstructed images of sizes 8 × 8 and 16 × 16 for lung phantom have been shown on left and right sides in Figure 5, respectively. We used the number of views as *p* = 18, for different cost functions (Table 1) to visually compare the reconstruction performance between 8 × 8- and 16 × 16-sized images using SA for the lung phantom. The original phantom image and cost functions have been implemented using the SA-sensitive parameters as follows (initial temperature T_*o*_ = 0.1, final temperature T_*N*_ = 1 × 10^–6^, annealing time N = 8 × 10^5^, temperature slab thickness set to 1,000 for which the temperature is kept constant to attain equilibrium in the image intensities, and temperature profile as given by Eq. 4). First two columns of Figure 5 show the results of the cost functions of 8 × 8-sized image. First image in first column is the original phantom (8 × 8) whereas the last image in the second column shows the best reconstructed image (8 × 8) corresponding to the RMSLE as the cost function. The other competing functions are RMSE and MAE in achieving good image quality. Some cost functions, such as UIQI, SSIM, and RSE, have not been found helpful in achieving good image quality. Similarly, for columns 3 and 4, the same trend has been observed for 16 × 16-sized image reconstruction whereas RMSLE has been found remarkable. The other competing cost functions that have produced outclass results are RMSE and MAE whereas rest of the cost functions seem requiring more annealing time to reach the acceptable image quality. Keeping in view the image quality with reduced number of views, significant reduction in radiation dose to the patients has been observed in case of lung biopsy sample images acquired using low-dose CT-guidance producing equivalent diagnostic accuracy images to standard dose CT-guidance (Arnold and Stahlecker, 2002).

**TABLE 3:** Comparison of cost functions for SA-based image reconstruction using performance measures: (PSNR, EuE, and WPSNR).

Image	Cost	Reconstructed image quality		
Size	Function	PSNR(dB)	EuE	WPSNR(dB)
8 × 8	UIQI	8.67 ± 0.72	1.06 ± 0.09	25.79 ± 0.92
	RMSE	24.20 ± 3.85	0.19 ± 0.06	62.59 ± 4.32
	SSIM	10.80 ± 0.90	0.83 ± 0.09	29.35 ± 2.11
	MAE	24.07 ± 2.80	0.18 ± 0.05	61.13 ± 7.39
	RSE	12.29 ± 0.98	0.7 ± 0.07	29.66 ± 2.15
	RAE	14.87 ± 1.19	0.51 ± 0.07	34.88 ± 1.83
	RMSLE	24.27 ± 3.10	0.18 ± 0.05	64.33 ± 1.98
16 × 16	UIQI	7.41 ± 0.27	1.19 ± 0.03	19.01 ± 0.82
	RMSE	26.28 ± 1.08	0.13 ± 0.01	68.11 ± 3.88
	SSIM	9.59 ± 0.49	0.93 ± 0.05	21.88 ± 0.89
	MAE	25.16 ± 1.43	0.15 ± 0.02	65.71 ± 3.13
	RSE	9.31 ± 0.42	0.96 ± 0.04	21.56 ± 0.97
	RAE	9.58 ± 0.49	0.93 ± 0.05	22.53 ± 0.71
	RMSLE	24.62 ± 1.15	0.16 ± 0.02	63.41 ± 1.87

**FIGURE 6:**
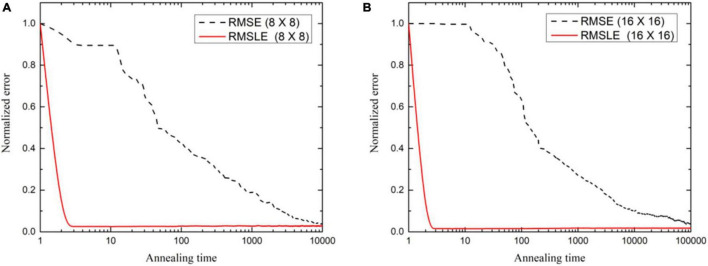
Convergence trends as normalized error variation against annealing time for **(A)** 8 × 8, and **(B)** 16 × 16 (*N* = 8×10^5^) lung phantom reconstruction using fan beam projections.

It may be said, in general, that larger-sized images need iterations to a greater extent to achieve an agreeable image quality. The image quality of the lung phantom is lower owing to the complexity of the reconstructed image. UIQI and SSIM, as cost functions, have been found to be requiring more annealing time to converge the reconstructed image to the same level as found with the other cost functions. This higher execution time is attributed to their inherent algorithmic complexity. In future, the decreasing computational cost per year for the same price may enable the use of appropriate cost functions by engaging parallel computing algorithms. The comparison of run times for numerous cost functions is shown in Table 4 for of 8 × 8- and 16 × 16-sized images. Keeping in view the image quality and also the run time comparison, RMSLE comes out to be the most appropriate choice. On the other hand, Figure 6 shows the convergence trends of RMSLE and RMSE cost functions for the lung phantom. The RMSLE converges efficiently in comparison with RMSE and it has been found that the former has been showing sharp error decline and less annealing time for 8 × 8 and 16 × 16 lung phantoms.

**TABLE 4:** Comparison of execution times for numerous cost functions for 8 × 8 and 16 × 16 lung phantoms.

Cost function	Run time (s)	
	Lung 8 × 8	Lung 16 × 16
UIQI	128.86 ± 3.44	613.74 ± 14.74
RMSE	21.73 ± 0.58	189.2 ± 10.02
SSIM	172.48 ± 3.73	924.29 ± 17.06
MAE	16.38 ± 0.53	191.19 ± 9.73
RSE	18.22 ± 0.59	148.16 ± 1.97
RAE	18.28 ± 0.94	157.76 ± 20
RMSLE	19.19 ± 0.31	147.8 ± 1.32

**FIGURE 7:**
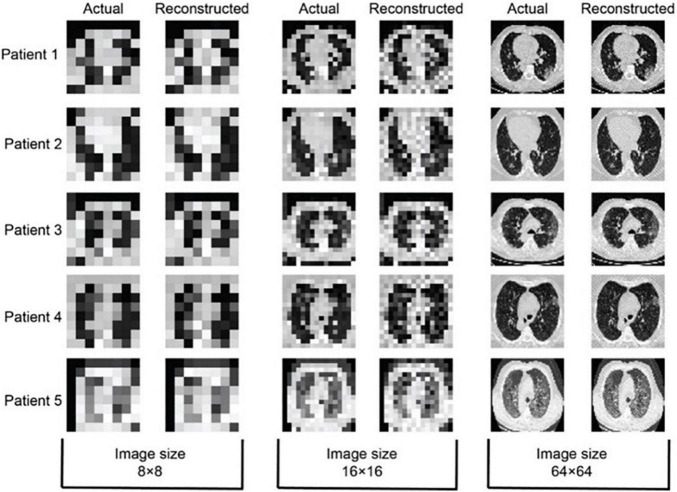
8 × 8, 16 × 16, and 64 × 64-sized COVID-19 reconstructed images using simulated annealing with RMSLE as cost function (*p* = 18, *T*_*0*_ = 0.1, *T*_*N*_ = 1 × 10^−6^, *N* = 2 × 10^5^, temperature slab thickness set to 1000, and temperature profile as given by Eq. 4).

Actual and reconstructed CT images of 8 × 8, 16 × 16, and 64 × 64 size of five confirmed patients with COVID-19 have been shown in Figure 7. The patients with COVID-19 have already low immunity and sensitive to X-ray radiation dose, so ultralow-dose CT has much importance in such cases (Agostini et al., 2020; Dangis et al., 2020) where the reduction in radiation exposure remains a topic of high interest. The most dose reduction approaches remained in the realm of decreasing tube current or tube voltage whereas iterative algorithms indemnify a satisfactory diagnostic image quality or a novel way to reduce radiation exposure to acquire less projection images. This compressed sensing (Candès et al., 2006; Donoho, 2006) is known as sparse-sampling CT. This approach allows acquiring a reduced number of projections for an additional dose reduction by a factor of two or more (Whiting et al., 2015; Mei et al., 2017). In the current case of patients with COVID-19, images are reconstructed by taking 18 sparse samples, so the reduction in dose is up to 10-fold. So, from concluding point of view the image quality, low-dose, and less-computational time comparison, RMSLE comes out to be a universal choice and has an efficient application for chest CT imaging of patients with COVID-19.

### Statistical Analysis

If a population has a normal distribution, the two prime activities of inferential statistics using sample data are the estimation of population mean, μ, and testing the claim about this population parameter (Triola et al., 2014). The confidence level (C), also known as confidence coefficient, is the probability (95% for experimentation) that the confidence interval actually does contain the population parameter (population mean), assuming that the estimation process is repeated a large number of times. The converse is true for significance level, α, representing the rejection region (two tails of the *t*-distribution). The analysis of our results has been conducted using Student’s *t*-test or the Mann–Whitney *U* test according to the normality tests. The confidence intervals for different cost functions using *t*-test have been illustrated in Table 5. The WPSNR mean values.

**TABLE 5:** Multiple resolution *t*-test analysis of cost functions based on 95% confidence interval (α = 0.05) and unknown population mean for sample size *n* = 20 with error margin **E** using **t_α/2_** = 2.093.

Resolution	Cost function	(AvgAcc)_*sample*_$\bar{\text{x}}$	(StdDev)_*sample*_ S	E	Confidence interval
8 × 8	UIQI	25.79	0.92	0.430568	25.36 < μ < 26.22
	RMSE	62.59	4.32	2.021799	60.57 < μ < 64.61
	SSIM	29.35	2.11	0.987499	28.36 < μ < 30.34
	MAE	61.13	7.39	3.458587	57.67 < μ < 64.59
	RSE	29.66	2.15	1.006219	28.65 < μ < 30.67
	RAE	34.88	1.83	0.856457	34.02 < μ < 35.74
	RMSLE	64.33	1.98	0.926658	63.40 < μ < 65.26
16 × 16	UIQI	19.01	0.82	0.383767	18.63 < μ < 19.39
	RMSE	68.11	3.88	1.815875	66.29 < μ < 69.93
	SSIM	21.88	0.89	0.416528	21.46 < μ < 22.30
	MAE	65.71	3.13	1.464868	64.25 < μ < 67.17
	RSE	21.56	0.97	0.453969	21.11 < μ < 22.01
	RAE	22.53	0.71	0.332286	22.20 < μ < 22.86
	RMSLE	63.41	1.87	0.875177	62.53 < μ < 64.29

$\bar{\text{x}}$, have been used along with the corresponding standard deviation, S, values (Table 3). A critical value, **t**_α/2,_ is borderline value separating sample statistics that are likely to occur from those that are unlikely to occur, whereas the margin of error or the maximum error of estimate, **E**=(tα_/2_S)/n, represent the variation between sample and population means. It can be interpreted that we are 95% confident that the interval from lower value to the higher value actually does contain the true value of population mean. If we were to experiment with multiple samples of size 20 and find the confidence intervals using *t*-test, 95% of them would contain the value of population mean.

In statistics, when a property of population has a claim, it is declared a hypothesis whereas a hypothesis test is a procedure devised to check this claim. The null hypothesis (H_0_) and alternative hypothesis (H_*a*_) for different cost functions have been illustrated in Table 6. The *t*-test statement for each of the cost function has been given in APA style guide. Since, *p*-value ≥ α, we accept the null hypothesis in each of the case. It can be inferred that for a 95% level of confidence, we accept the null hypothesis (${\text{H}}_{0}:\mu =\bar{\text{x}}$) that the mean WPSNR value is representing the population mean. So, with 95% confidence, we believe that there is no evidence to reject the null hypothesis.

**TABLE 6:** Multiple resolution *t*-test statement in APA style for different cost functions used for image reconstruction (two-tailed *t*-distribution with no inequality in alternate hypothesis), the null hypothesis is ${\text{H}}_{0}:\mu =\bar{\text{x}}$, and the alternative hypothesis is ${\text{H}}_{\text{a}}:\mu \neq \bar{\text{x}}$.

Resolution	Cost function	(AvgAcc)_*sample*_$\bar{\text{x}}$	(StdDev)_*sample*_ S	*t*-test statement
8 × 8	UIQI	25.79	0.92	t(19) = 2.093, *p* = .05
	RMSE	62.59	4.32	
	SSIM	29.35	2.11	
	MAE	61.13	7.39	
	RSE	29.66	2.15	
	RAE	34.88	1.83	
	RMSLE	64.33	1.98	
16 × 16	UIQI	19.01	0.82	
	RMSE	68.11	3.88	
	SSIM	21.88	0.89	
	MAE	65.71	3.13	
	RSE	21.56	0.97	
	RAE	22.53	0.71	
	RMSLE	63.41	1.87	

### Evaluation Based on Comparative Performance

We have implemented the FBP (Hansen et al., 2021) and algebraic reconstruction technique (ART) (Andersen, 1989) for comparison with the proposed methodology using patients with COVID-19. The experimentation has been carried out using *p* = 18 as the uniform basis with their published parametric set. The experimental results have been illustrated in Table 7. It has been found that the RMSLE-based image reconstruction using SA appears promising in comparison with FBP and ART methods using limited number of projections (Section “Wavelet Transform-Based Initializing Template”).

**TABLE 7:** Comparison of proposed cost function-based reconstruction with other works for 8 × 8-, and 16 × 16-sized square images using lesser number of projections (*p* = 18).

No.	Method	8 × 8			16 × 16		
		PSNR (dB)	EuE	WPSNR (dB)	PSNR (dB)	EuE	WPSNR (dB)
Patient 1	FBP	+9.45	1.08	+15.64	+11.77	0.82	+21.34
	ART	+14.71	0.52	+33.67	+14.71	0.51	+33.67
	This work	+24.32	0.14	+55.46	+20.52	0.28	+65.58
Patient 2	FBP	+9.22	0.98	+13.22	+11.98	0.79	+21.79
	ART	+13.72	0.57	+29.39	+13.72	0.57	+29.39
	This work	+26.91	0.13	+58.77	+20.93	0.29	+64.35
Patient 3	FBP	+9.11	0.96	+13.10	+12.11	0.77	+21.22
	ART	+13.64	0.56	+25.66	+13.64	0.56	+25.66
	This work	+25.65	0.16	+56.55	+22.51	0.21	+63.65
Patient 4	FBP	+9.37	0.97	+13.02	+12.02	0.81	+21.37
	ART	+14.39	0.57	+25.57	+14.39	0.57	+25.57
	This work	+23.91	0.22	+52.14	+21.52	0.26	+64.08
Patient 5	FBP	+9.83	0.91	+16.32	+12.07	0.79	+21.23
	ART	+15.07	0.51	+34.05	+15.07	0.51	+34.05
	This work	+27.35	0.13	+61.23	+21.65	0.25	+62.19

We have achieved ultralow-dose X-ray CT of patients with COVID-19 by stochastically estimating images. The sensitivity analysis of different cost functions in terms of computational and spatial complexity has been performed using image quality measures, namely PSNR, EuE, and WPSNR. We have reconstructed chest CT images of patients with COVID-19 using RMSLE with eighteen projections. This led to 10-fold reduction in radiation dose exposure which may help for accurate diagnosis of patients with COVID-19 with less immunity and sensitive to radiation dose. Some of the demerits in this research activity may be enumerated as the high run time required that is generally associated with stochastic processes. In addition to this, extensive parallelism is required for large-sized images to get the results in adequate time. The shape complexity of the image to be estimated is another important factor that can affect the convergence trend.

## Conclusion

The objective of this work is to introduce ultralow-dose X-ray CT methods along with a suitable cost function for early and reliable diagnosis of elderly and also individuals subjected to the pandemic with dire consequences. First, we have applied the ultralow dose rate SA on lung phantom reconstruction going through different cost functions, and then actual reconstruction is carried out using real patients’ CT scan. The cost function analysis for image reconstruction using SA has been carried out to compare the improvement in the image quality and their convergence trends. The numerous cost functions used are UIQI, RMSE, SSIM, MAE, RSE, RAE, and RMSLE. For the same set of parameters, RMSLE generally is considered outperforming relatively. The UIQI and SSIM are classified as the subjective image quality measures that will be promising in near future where more annealing time is relatively reasonable. For 8 × 8 and 16 × 16 lung phantoms, RMSLE cost function has resulted in WPSNR of 64.33 ± 3.98 dB and 63.41 ± 2.88 dB, respectively. So, RMSLE can be implemented to reconstruct the chest CT images of patients with COVID-19. A comprehensive comparison of existing reconstruction techniques shows that using only eighteen number of projections, a 10-fold reduction in radiation dose exposure is a need to adopt computer-aided diagnostic techniques as a second opinion with expert advice.

## Data Availability Statement

The raw data supporting the conclusions of this article will be made available by the authors, without undue reservation.

## Author Contributions

SQ and AR conceived of the presented idea. SQ developed the theory and supervised the findings of this work. AR, AM, MR, and WM validated the theory and performed the computations. SQ, AR, AM, MR, and WM verified the methods. All authors discussed the results and contributed to the final manuscript.

## Conflict of Interest

The authors declare that the research was conducted in the absence of any commercial or financial relationships that could be construed as a potential conflict of interest.

## Publisher’s Note

All claims expressed in this article are solely those of the authors and do not necessarily represent those of their affiliated organizations, or those of the publisher, the editors and the reviewers. Any product that may be evaluated in this article, or claim that may be made by its manufacturer, is not guaranteed or endorsed by the publisher.
